# 2745. *In-Vitro* Activity of Cefiderocol Against Multidrug Resistant Strains of *P. aeruginosa* DTR, *A. baumannii* complex DTR, *A. xylosoxidans* MDR, *S. maltophilia* MDR & *B. cepacia* complex, by Iron-Depleted Broth Microdilution

**DOI:** 10.1093/ofid/ofad500.2356

**Published:** 2023-11-27

**Authors:** Jose Alexander, Daniel Navas, Angela Charles

**Affiliations:** Advent Health Central Florida, Orlando, Florida; AdventHealth Orlando, Orlando, Florida; AdventHealth, Orlando, Florida

## Abstract

**Background:**

The emergence of multidrug-resistant (MDR) & difficult-to-treat (DTR) non-fermenter (NF) organisms poses a critical challenge for antimicrobial therapy, threatening the efficacy of existing antimicrobial and contributing to an increasing worldwide mortality rate. MDR NFs are among the most challenging organisms isolated in the clinical laboratory. Cefiderocol (CFD), a novel siderophore cephalosporin, offers an interesting approach by exploiting the bacteria's iron-acquisition system and its intrinsic stability against multiple classes of β-lactamases. In this descriptive abstract, we present the *in-vitro* activity of cefiderocol against multiple strains of NF organisms.

Tab.1

**Figure:**
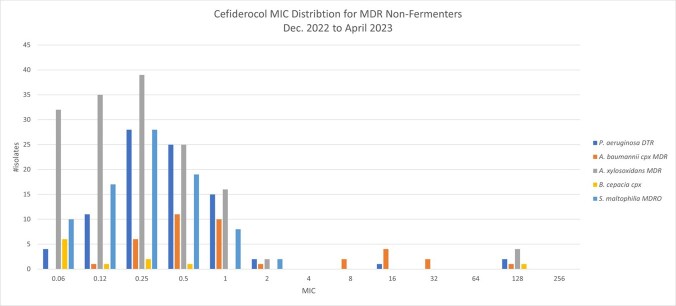

Cefiderocol MIC distribtion for MDR non-fermenters. Dec. 2022 to April 2023

**Methods:**

MIC values by iron-depleted broth microdilution (Liofilchem ComASP®) were collected and distributed by organism from December 2022 up to April 2023 at AdventHealth Orlando Microbiology Department. *P. aeruginosa* DTR (n=88), *A. baumannii* cpx DTR (n=38), *A. xylosoxidans* MDR (n=153), *S. maltophilia* MDR (n=84) & *B. cepacia cpx* (n=11). *P. aeruginosa* DTR is defined as an isolate intermediate (I) or resistant (R) to all tested antimicrobials except aminoglycosides. *A. baumannii* DTR as an isolate I or R to ampicillin/sulbactam and meropenem and one more active agent. *A. xylosoxidans* MDR as an isolate I or R to one agent in at least three active antimicrobial classes & *S. maltophilia* MDR as an isolate I or R to trimethoprim/sulfamethoxazole.

**Results:**

The average, mode, median and range MIC (µg/mL) were calculated for each organism. *P. aeruginosa* DTR: 3.55, 0.25, 0.5, 0.06-128; *A. baumannii* DTR: 7.66, 0.5, 0.5, 0.12-128; *A. xylosoxidans* MDR: 3.66, 0.25, 0.25, 0.06-128; *S. maltophilia* MDR 0.37, 0.25, 0.25, 0.06-2; *B. cepacia* cpx: 11.77, 0.06, 0.06, 0.06-128.

**Conclusion:**

Using the *P. aeruginosa* susceptible CLSI BP (< =4 µg/mL) as a reference for *P. aeruginosa* DTR, *A. xylosoxidans* MDR & *B. cepacia* cpx, 96.6%, 97.39% & 90.9% respectively have an MIC value in the susceptible BP. 76.3% of *A. baumannii* DTR have an MIC < =4 µg/mL (susceptible BP) and 97.6% of *S. maltophilia* MDR have a susceptible CLSI BP < =1 µg/mL. Although clinical outcome data is needed, cefiderocol demonstrated a significant potency against the most resistant NF strains and may represent a significant therapeutic option for difficult to treat isolates.

**Disclosures:**

**Jose Alexander, MD, D(ABMM), FCCM, CIC, SM/MB(ASCP), BCMAS**, Shionogi: Advisor/Consultant|Shionogi: Honoraria

